# Joint modelling of colorectal cancer recurrence and death after resection using multi-state model with cured fraction

**DOI:** 10.1038/s41598-020-79969-6

**Published:** 2021-01-13

**Authors:** Behnaz Alafchi, Ghodratollah Roshanaei, Leili Tapak, Mohammad Abbasi, Hossein Mahjub

**Affiliations:** 1 Department of Biostatistics, School of Public Health, Hamadan University of Medical Sciences, Hamadan, Iran; 2 Department of Biostatistics, School of Public Health, Modeling of Noncommunicable Disease Research Center, Hamadan University of Medical Sciences, Hamadan, Iran; 3 Department of Biostatistics, School of Public Health, Modeling of Noncommunicable Diseases Research Center, Hamadan University of Medical Sciences, Hamadan, Iran; 4 Faculty of Medicine, Shahid Beheshti Hospital, Hamadan University of Medical Sciences, Hamadan, Iran; 5 Department of Biostatistics, School of Public Health and Research Center for Health Sciences, Hamadan University of Medical Sciences, Hamadan, Iran

**Keywords:** Diseases, Gastroenterology, Medical research, Risk factors

## Abstract

Curing of colorectal cancer (CRC) occurs at the time of resection but it is not immediately observable. If the cancer is not completely eliminated, the patient will not be cured of cancer and will experience recurrence as the tumor has regrown to a detectable size. The main propose of the present study was to assess the effects of different covariates on the probability of being cured as well as the time-to-recurrence, and time-to-death in CRC patients by using multi-state cure model. The information of 283 patients with CRC, who underwent resection, from 2000 to 2015 in Imam Khomeini Hospital of Hamadan, Iran, were analyzed. The results of multi-state cure model reveal that females and who experience metastasis were more likely to be apparently cured. It has been shown that sex has a significant effect on the time-to-recurrence given patient was in the not cured group. The survival time of patients of the not cured group was affected by the stage of disease. However, the survival of the apparently cured patients were affected by age at diagnosis and metastasis status. The multi-state cure model provided a flexible framework to study the effects of prognostic factors simultaneously on the transition between different states and the probability of being apparently cured of CRC.

## Introduction

Colorectal cancer (CRC) is a major cause of mortality and morbidity. It is one of the most commonly diagnosed cancer^1^, and the fourth common cause of cancer-related death worldwide^2,3^. The CRC survival is highly dependent upon the stage of disease at diagnosis as well as the possibility of resection of the tumor^4,5^.

The only curative treatment for CRC patients is complete surgical resection. About 70–80% of patients are eligible for curative resection^6,7^. Moreover, almost two-thirds of CRC patients underwent resection but 30–50% of these patients will experience recurrence and will die of CRC^6,8^. However, curing happens at the time of treatment, it is not immediately observable. If cancer cannot be completely eliminated, the patient will not be cured and will experience a recurrence as the tumor has regrown to a detectable size^9^. Patients who experience a recurrence are substantially at higher risk of mortality and it is essential to find out which prognostic factors predispose a patient to recurrence^10–14^. The time of occurrence of recurrence also influences the overall survival, such that worse survival is associated more with the early recurrence than the late one^15^.

Recurrence can modify the cancer progression and the effects of prognostic factors may have be changed by recurrence. So, the effects of variables on the time of recurrence, the survival time and the recurrence-free survival time may be different. Therefore, especial statistical methods are required to obtain such effects^16,17^. Various analytic methods can be used to analyze the time of recurrence or time-to-death in such settings^11,12,18–20^. Usually, separate survival analyses are carried out for these clinical events. Nevertheless, these separate analyses are not completely satisfying because they may fail to reveal the relationships between recurrence and death event^21,22^. On the other words, a patient may experience a clinical progression (e.g. a local recurrence, followed by a metastatic recurrence and then death), so instead of the occurrence of a single event, the progression of disease should be modeled jointly.

A common way to joint modeling of different types of events is to use multi-state models, which describe the progression of the disease and transitions between different states over time. In this model, each event or each transition between events is considered as a disease state^23^. Multi-state models are usually specified by using transition intensities and can be based on two scales of time including: the calendar time and the duration time in the current state, called Markov and semi-Markov model, respectively. In fact, in the Markov models, $t_{0} = 0$ is taken as the entry time and the other subsequent times are referred to the time since $t_{0}$^24^. Moreover, Markov model assumes that the future evolution of the system depends on the history solely through the current state. While in the semi-Markov model the clock is set to back zero at the time of entry in a new state. Moreover, in this type of model, it is assumed that the future state is dependent not only on the current state but also on the sojourn time in the current state^25,26^. On the other hand, as some patients may be apparently cured after treatment and will never experience recurrence of CRC, cure models should be used.

Cure models are used to model many different types of diseases when a substantial proportion of patients are completely cured by the treatment and will never experience the clinical recurrence^27^. A multi-state cure model is a multi-state model which incorporates a latent cured state and combines the aspects of both multi-state and cure model to investigate the effects of the covariates on both curing of the disease and the disease processes as well as dealing with the association between different events of interest (recurrence/death), simultaneously^28^. Although, the progression of CRC disease includes different states and a fraction of patients apparently cured after the resection, there has not yet been conducted any study that analyzes CRC data using this model. So, in this study, a multi-state cure model is used to joint modeling of the recurrence and death in patients who developed CRC and underwent curative resection, considering the probability of being apparently cured after resection.

## Results

**Figure 1:**
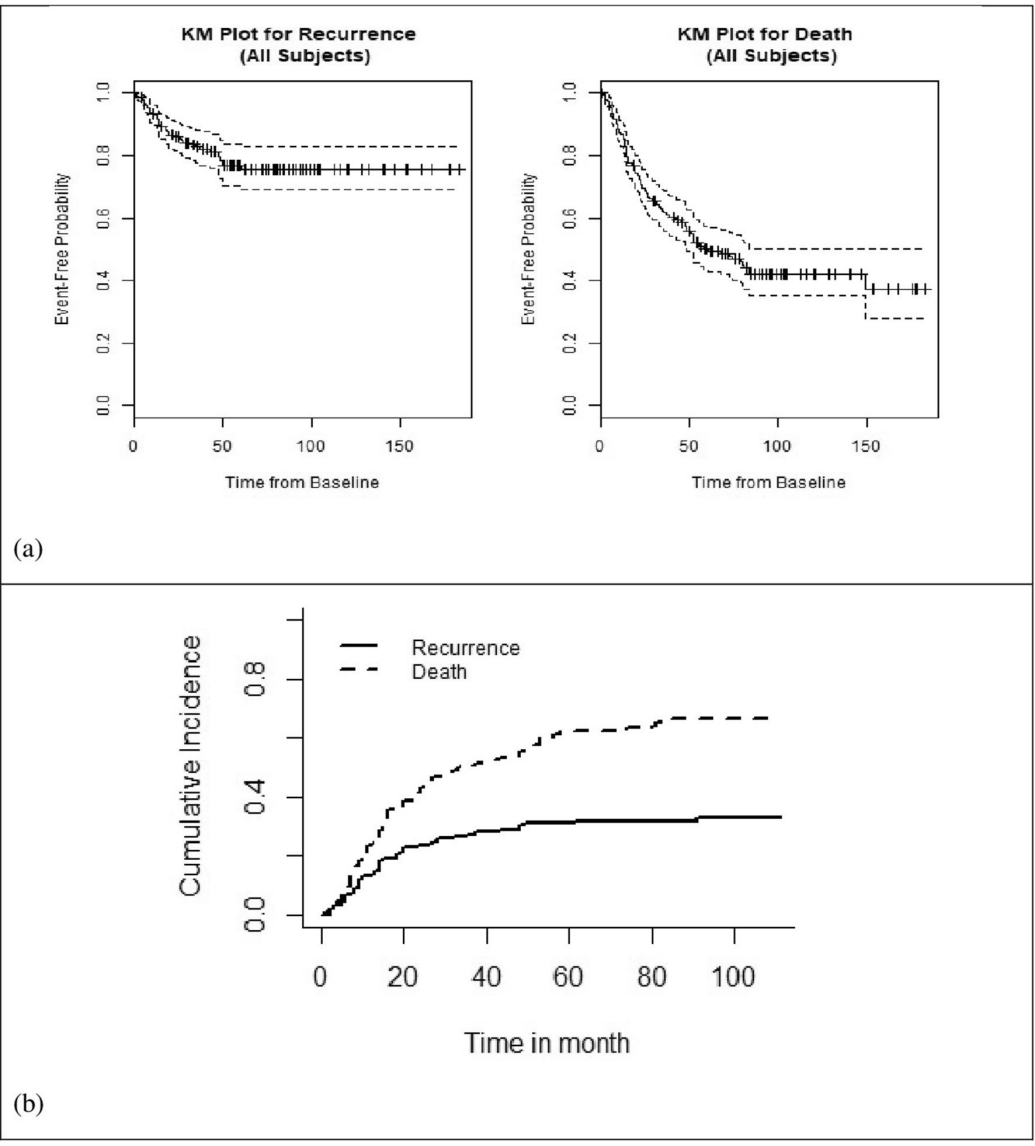
(**a**) Kaplan–Meier (KM) survival curves for recurrence and death times; (**b**) cumulative incidence for recurrence and death after resection.

Of 283 patients underwent curative resection for CRC, 99 (35%) patients had rectal and 184 (65%) patients had colon adenocarcinomas. The frequency of CRC in both male and female sexes was almost the same (52.7% female and 47.3% male) with mean age of 55.58 ± 13.127. For more than 90% of patients, surgery was the first treatment that they received. About 67 (23.7%) patients were diagnosed with metastasis and 44 patients developed metastasis during their follow up period. Overall, 40 percentage of the patients had metastatic CRC (45.5%, 9.1%, 8.2%, and 37.2% of metastasis were in liver, lung, lung and liver, and other tissues, respectively). All of the patients were diagnosed being at more advanced stages such that none of them were diagnosed at stage I (132 (46.6%) at the stage II, 84 (29.7%) at the stage III, and 67 (23.7%) at the stage IV). The number of patients who have received chemotherapy and radiotherapy after resection were 242 (85.5%) and 89 (31.4%), respectively. Duration, frequency, type/dose of drug, and the number of cycles of chemotherapy (ranged from 1 to 39 by mean of 6.41 ± 4.6 session) were different among patients. The mean of BMI for the subjects was 22.21 ± 3.83. During the study, 44 (15.5%) patients experienced recurrence after resection. Figure 1a shows the Kaplan–Meier survival curve for recurrence and death. According to this figure, Kaplan–Meier plot of all the data showed a clear plateau for recurrence which justifies the use of cure models. However, the Kaplan–Meier plot for recurrence takes death before recurrence as non-informative censoring, so we also provided the cumulative incidence for recurrence and death after resection in Fig. 1 part (b).

**Figure 2:**
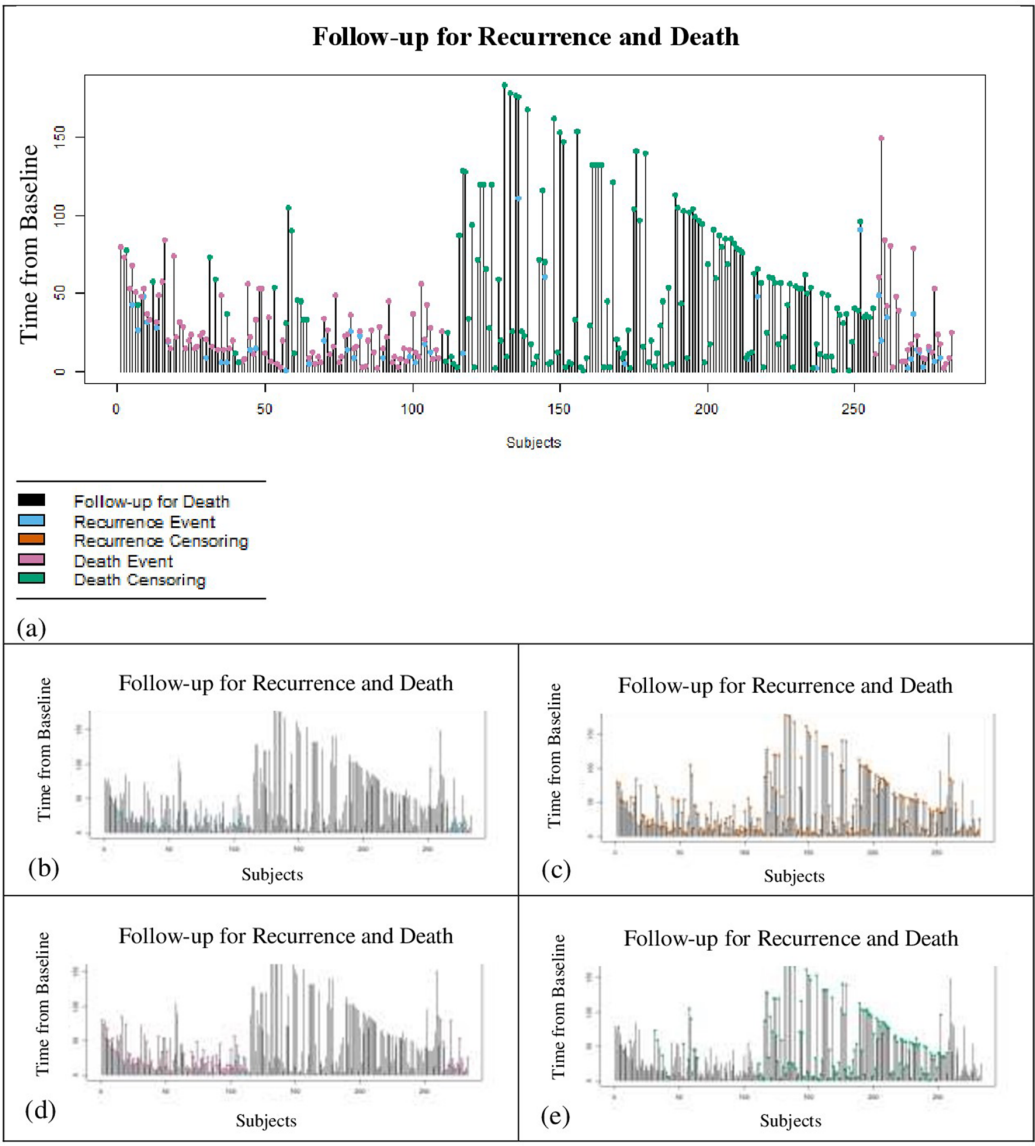
Length of follow-up for recurrence and death events. The lines represent the follow-up time. Part (**a**) display the overall time for all patients. The other parts show separately (**b**) the time of recurrent event (blue dots), (**c**) the time of censoring for recurrent event (orange dots), (**d**) the time of death event (pink dots), and (**e**) the time of censoring for death (green dots).

The lines in Fig. 2a depict the follow-up times of overall survival for each subject. The events and censoring times for both recurrence and death (dots) were also shown in Fig. 2 (Fig. 2b-e), indicating that there was not unequal censorship. So, we assumed the censoring times for both events (recurrence and death) were equal. It could be seen that the majority of the observed recurrences occurred early in follow-up and were slowed down substantially by about 50 months (Fig. 2a). Therefore, it seemed reasonable to consider patients who were still at risk for recurrence and death after 50 months as apparently cured patients.

The results of multi-state cure model were provided in Table 1 which shows the effects of the variables including age at diagnosis, sex, metastasis, stage of the disease at diagnosis, and chemotherapy on the hazards of different transitions between various states as well as on the probability of being not cured of CRC after resection. It should be noted, as all of the patients who were not cured of CRC received chemotherapy, it was not possible to assess the effects of chemotherapy on the survival time of these patients.

**Table 1:** Results of fitting multi-state cure model to the colorectal cancer data.

Variable	Coefficient (B)	SE	OR (e^B^)	0.95% CI (B)	p-value
**(A) The probability of not being cured (logistic model)**					
Intercept	1.241	0.72	3.459	(− 0.17, 2.65)	0.318
Age	0.046	0.03	1.047	(− 0.01, 0.10)	0.077
Sex (ref: female)	− 0.811	0.41	0.444	(− 1.60, − 0.02)	0.045*
Stage III (ref: stage II)	0.194	0.54	1.214	(− 0.87, 1.25)	0.719
Stage IV (ref: stage II)	0.309	0.48	1.362	(− 0.64, 1.26)	0.522
Chemotherapy (ref: no)	0.539	0.37	1.714	(− 0.18, 1.26)	0.145
Metastasis (ref: no)	1.468	0.33	4.343	(0.82, 2.11)	< 0.001*

Variable	Coefficient (B)	SE	HR (e^B^)	0.95% CI (B)	p-value
**(B) Apparently cured to death (Transition 1 → 4)**					
Age	0.076	0.03	1.079	(0.02, 0.14)	0.015*
Sex (ref: female)	0.321	0.62	1.378	(− 0.89, 1.53)	0.603
Stage III (ref: stage II)	0.799	0.64	2.224	(− 0.45, 2.05)	0.210
Stage IV (ref: stage II)	1.287	0.77	3.624	(− 0.22, 2.79)	0.093
Chemotherapy (ref: no)	− 1.035	0.58	0.355	(− 2.17, 0.10)	0.075
Metastasis (ref: no)	2.003	0.79	7.411	(0.46, 3.54)	0.011*
**(C) Not cured to death (Transition 2 → 4)**					
Age	0.013	0.32	1.013	(− 0.05, 0.08)	0.688
Sex (ref: female)	0.059	0.71	1.061	(− 1.33, 1.45)	0.933
Stage III (ref: stage II)	2.583	0.99	13.242	(0.64, 4.53)	0.009*
Stage IV (ref: stage II)	2.651	0.88	14.154	(0.91, 4.39)	0.003*
Metastasis (ref: no)	0.297	0.42	1.346	(− 0.53, 1.12)	0.479
**(D) Not cured to recurrence (Transition 2 → 3)**					
Age	0.031	0.02	1.031	(− 0.02, 0.08)	0.215
Sex (ref: female)	1.083	0.51	2.953	(0.08, 2.08)	0.034*
Stage III (ref: stage II)	0.411	0.85	1.509	(− 1.26, 2.09)	0.631
Stage IV (ref: stage II)	0.831	0.89	2.293	(− 0.92, 2.58)	0.352
Chemotherapy (ref: no)	− 0.091	0.73	0.913	(− 1.52, 1.34)	0.901
Metastasis (ref: no)	− 0.486	0.82	0.615	(− 2.10, 1.13)	0.555
**(E) Recurrence to death (Transition 3 → 4)**					
Stage III (ref: stage II)	0.936	0.56	2.551	(− 0.16, 2.03)	0.093
Stage IV (ref: stage II)	1.030	0.60	2.802	(− 0.16, 2.22)	0.089
Metastasis (ref: no)	0.466	0.59	1.594	(− 0.68, 1.62)	0.427
Time-to-recurrence	− 0.005	0.01	0.994	(− 0.03, 0.02)	0.674

*Significant at 0.05; ref = reference level.

As the results showed (Table 1A), sex and experiencing metastasis had a significant effect on the probability of not being cured, such that females (p = 0.045), and patients with metastasis (p < 0.001) were less likely to be cured of CRC. The hazard of death was greater in apparently cured patients who experienced metastasis (p = 0.011). The hazard of death also increased with an increase in the age, given patient was in the apparently cured group (p = 0.015) (Table 1B). The risk of death for not cured patients increased substantially by diagnosing the disease at more advanced stages, as patients at stage III (p = 0.009) and stage IV (p = 0.003) compared with patients at the stage II were at higher risk of death (Table 1C). The risk of recurrence was about 3 times higher in male, given patient was in the not cured group (p = 0.034) (Table 1D). The effects of variables on different parts of the multi-state cure model are shown in Supplementary Fig. A in the Supplementary Appendix.

**Figure 3:**
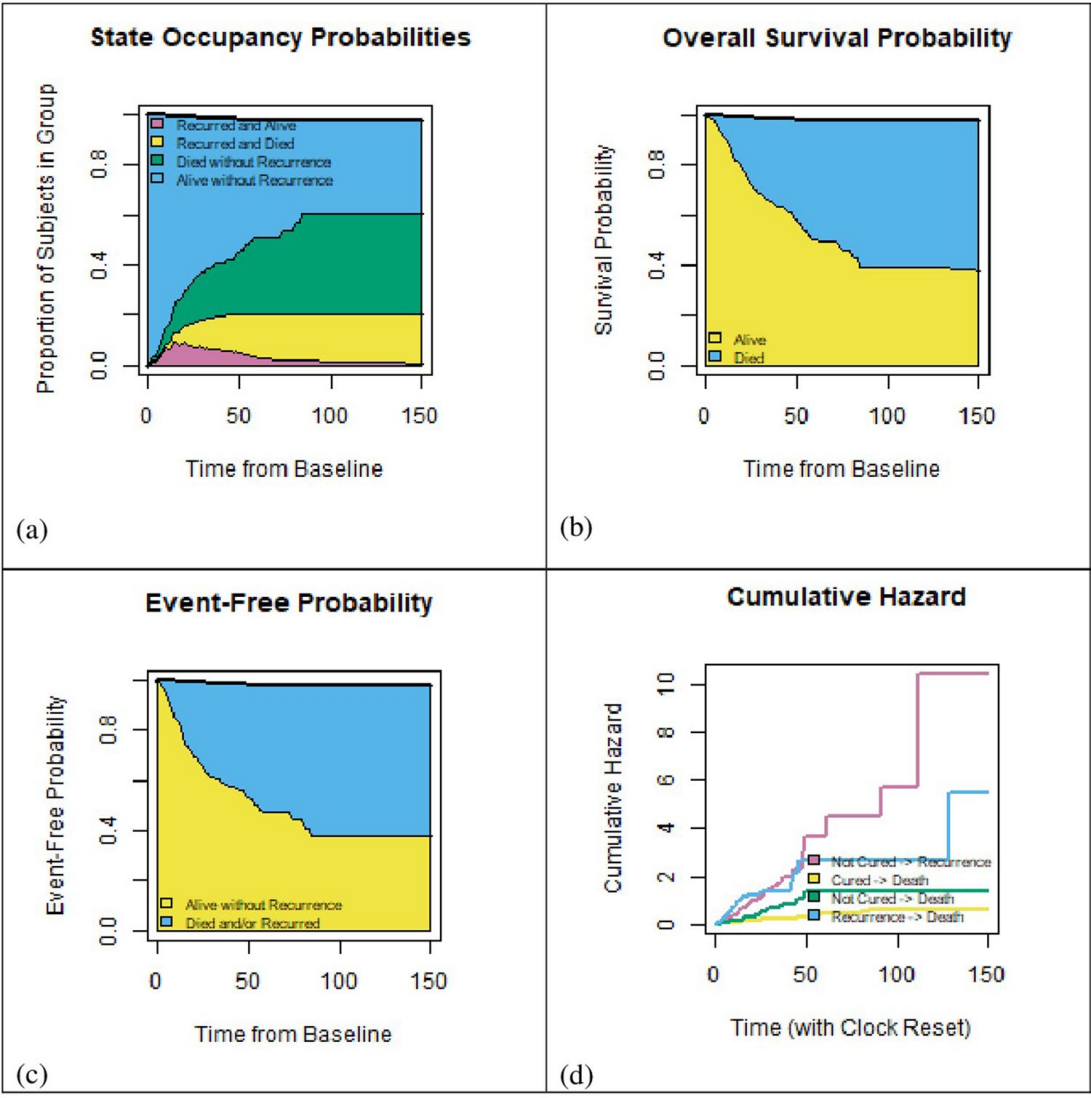
State occupancy probabilities, overall survival, event-free survival, and cumulative hazards for all transitions according to Multi-state cure model for a hypothetical subject with mean values of all covariates.

Figure 3 shows the state occupancy probabilities, overall survival probability, event-free probability and cumulative hazards for a hypothetical subject with mean values of all covariates. It should be noted that in this figure the effect of time-to-recurrence as a covariate was not taken into account on the transition from recurrence to death. Figure 3 part (a) shows the state occupancy probabilities (constructed by the probability that an individual be in a specific state at any given time) in which the horizontal axis represents time in months and the vertical axis shows the cumulative probability of being in a particular state. It can be seen that the risk of death has increased over time either the patient was in apparently cured or in not cured group. It also exhibited that such patient was most likely to did not experience the recurrence and was alive at the end of the follow-up. The overall survival (the time from treatment until death or censoring at the last follow up) an event-free survival (the time from treatment to recurrence or death whichever occurs first) were two interested endpoints. Figure 3b,c show these two survival probabilities at different times for given patient. The cumulative hazards for such hypothetical patient, for all four transitions, are also presented in Fig. 3 part (d).

## Discussion

In the present study, a multi-state semi-Markov model was used to joint modeling of recurrence and death in colorectal cancer (CRC) with an incorporated cured fraction, in order to study the factors that influence the transition intensities between different states. The structure of this multi-state cure model was motivated by the disease process of CRC. This model was first introduced by Conlon et al. in 2014 to analyze colon cancer data^28^ and Lauren et al. in 2018 extended an EM algorithm to estimate the parameters of this model and applied their model on head and neck cancer data^29^. As we were awarded, there were no study that have applied this model on CRC data and assessed the effects of variables on recurrence and death events jointly in the presence of cured fraction of patients.

It has been reported that 30–50% of CRC patients who underwent resection will experience the recurrence^6,8^. Although, our results revealed that the tumor of a significant proportion of patients was eliminated by the treatment so that they will never experience a recurrence of CRC. Moreover, as there was a sufficient follow-up period and a number of patients who were censored for recurrence after the last observed time (the Kaplan–Meier survival plot for recurrence event in Fig. 1a shows a clear level plateau), it was justifiable to use a mixture cure model for the recurrence event^27^. On the other hand, as the recurrence and death events were correlated, joint modeling of recurrence and death events could diminish the bias which might occur in separated model.

The joint modeling of the recurrence and survival time could also aid in identifiability of the cure part of the model because subjects with survival greater than the last observed recurrence time were likely to be apparently cured of the recurrence^28^.

According to the findings, there was a significant association between sex and having metastatic tumor with the probability of being cured. The result suggested that females were less likely to be cured of CRC (1/OR = 1/0.444 = 2.252: the odds of being cured in males was 2.252 times of females). The results also suggested that the patients who had metastatic tumor were less likely to be cured of CRC (1/OR = 1/4.343 = 0.230: the odds of being cured in patients who experience metastasis was 0.230 times less than others). However, the effect of the variables on the recurrence of CRC have been assessed in other studies^30–32^, we have not found any study that have investigated its effect on the probability of being cured.

Generally, in patients with CRC, death can occur with or without a prior recurrence. The deaths following a recurrence may be due to the cancer, whereas the deaths without a prior recurrence are known not to be directly due to the regrowth of the tumor^28^. However, the cause of death was not considered in this study and we have not followed this issue as a competing risk event.

The results showed that the survival time of patients after resection was affected by age at diagnosis and metastasis in those that their cancer was completely eliminated by resection (apparently cured patients), and stage of the disease in those who may experience the recurrence lately (not cured patients). We did not expect that the metastasis be affective on the survival time of apparently cured patients. It should be noted that these patients were apparently cured of CRC and they had no sign of recurrence. Detailed examination of the data revealed that just 26% of patients in apparently cured state were diagnosis with metastasis (none of them developed metastasis during their follow up) but about 80% of these patients were died by the end of study. It seems that a larger sample size was needed to better look at this finding. Pagès et al. showed that early metastatic invasion can decrease the survival time of CRC patients^33^. Other studies also indicated that patients who had metastasis to other tissues were at higher risk of death after surgery^34,35^.

It also can be concluded from the findings, as the age of patients increased, the hazard of death in apparently cured patients increased as well. The effect of age on the survival of CRC patients had been assessed in different studies which are controversy. Some of them indicated that older patients are at higher risk of death^19,36,37^, while the results of some others did not show a significant association between age and the risk of death in CRC patients^38–41^. However, none of these studies assessed the effect of age separately on the survival rate of apparently cured and not cured CRC patients.

Moreover, it has been shown that the risk of death was substantially higher in patients diagnosed with more advanced stages (patients at stage IV and III were at higher risk of death compared those at stage II). It should be noted that stage of the disease at diagnosis were just significantly effective on the survival time of patients whose disease was not cured by resection and the tumor of more than 50% of these patients, was regrowth. Other studies also showed a significant association between the stage of the CRC at diagnosis and the survival time^33,39,42,43^.

On the other hand, two competing risks (recurrence/death) were encountered by the patients after resection. Among 30% (85 of 283) of patients who were not cured by resection, 51.8% (44 of 85) experienced the recurrence. The results showed that the odds of being cured were higher in males (based on the logistic part of the model). On the other hand, males were at higher risk of recurrence after resection by HR = 2.953, given patient was in the not cured group. Tartter^31^ and Kobayashi et al.^44^ have showed that the risk of recurrence is significantly different in both males and females patients with colon and rectal cancer while based on the survival analysis there were no association between sex and recurrence time or disease-free survival. Dancourt et al.^19^ by joint modeling of recurrence and death in CRC data using a multi-state model showed that the time of recurrence is affected by sex and males were more likely to be recurred.

The results also showed that the risk of death after recurrence among patients who were diagnosed at stages III and IV, were 2.55 and 2.80 times of the patients who were at stage II, respectively. However, these effects were not statistically significant. Other clinical study also had showed that the patients who underwent resection and diagnosed at stage III had a greater probability of death after experiencing recurrence than the patients at stage I&II^19^.

The results also revealed that chemotherapy did not have a substantial effect on any transition intensities. As most of our patients underwent chemotherapy, assessing the effect of this variable were not possible in two transitions. Chemotherapy schedule were different. Most of studies have assessed the effect of chemotherapy on the rate and time of (local) recurrence after resection^6,45–48^. Collaborative Group showed that the relative risk of recurrence and death were higher in patients underwent chemotherapy. However, according to their findings there was no significant difference in efficacy of treatment by chemotherapy schedule^47^.

This study has some limitations. First, for survival analysis, reliable data based on prospective cohort studies are required. However, our data was based on a retrospective study and information was based on the data recorded by registry centers. Therefore, we were unable to assess the accuracy of the data. This issue may introduce information bias. Moreover, according to this limitation some important variables such as period/exposition to chemotherapy/radiotherapy and clinical state of the patients were not included in the collected data. Second, although patients were followed about 15 years, the number of all available patients who underwent resection was limited. On the other hand, as in the multi-state cure model, there were many parameters to be estimated and their number increased by the number of variables in each transition, our sample size was relatively small. Due to this limitation, the confidence interval of some HR was relatively wide. It is clear that bigger sample sizes will provide much more precise estimates. Third, in such studies, the censored times for death and recurrence are not necessarily equal as the recurrent time needs an active follow-up while death/alive status (death obtaining) can be obtained at a later time. In the other words, recurrence can only be ascertained at a discrete evaluation time. However, some researchers have proposed estimation methods for the illness-death model under this type of dual censoring^49^, this is yet to be extended to the multi-state cure model.

Despite these limitations, the main purpose of the present study was to use powerful statistical methods (here multi-state cure model) which takes several aspects of the data into account. In the future clinical studies, it is suggested that if there were different states of disease, such multi-state cure model would be used instead of separated models to analyze the data.

## Conclusions

The multi-state cure model provided a flexible framework to study and compare the effects of prognostic factors simultaneously on transition between different health states and the probability of being cured of CRC. In summary, the results revealed that females and who experience metastasis were more likely to be apparently cured and more than 50% of not cured patients recurred later. Furthermore, the survival time of the CRC patients after resection was affected by stage of the disease at diagnosis, age and metastasis. Also, the time-to-recurrence of CRC was affected by the sex of patients.

## Methods

### Ethical approval

This study was performed after receiving approval from the Ethics Committee of Hamadan University of Medical Sciences and was conducted with confidentiality regarding patients’ name and surname. All study participants, or their legal guardian, provided informed written consent prior to study enrollment. All methods were performed in accordance with the relevant guidelines and regulations.

### Description of dataset

The information of 283 patients with adenocarcinoma CRC who underwent curative resection and admitted to Imam Khomeini Hospital in Hamadan in the west of Iran, between 2000 and 2015 were analyzed in this study. Patients were followed to August 2017. The information of vital status and date of death was obtained through medical and administrative recorded sources. Here, all deaths were considered as CRC-related deaths. The information of baseline demographic and clinical variables including sex (male/female), age at diagnoses (year), body mass index (BMI), metastasis (yes/no), stages of the disease based on TNM^50^ classification (stage I/stage II/stage III/stage IV), receiving chemotherapy and/or radiotherapy (yes/no), were collected from medical records. The outcomes of interest were time of entry in each state including time to recurrence, time to death and time to death after recurrence. All patients who were alive at the end of study were censored for death and who did not experience recurrence of CRC were also censored for recurrent event.

### Multi-state cure model

**Figure 4:**
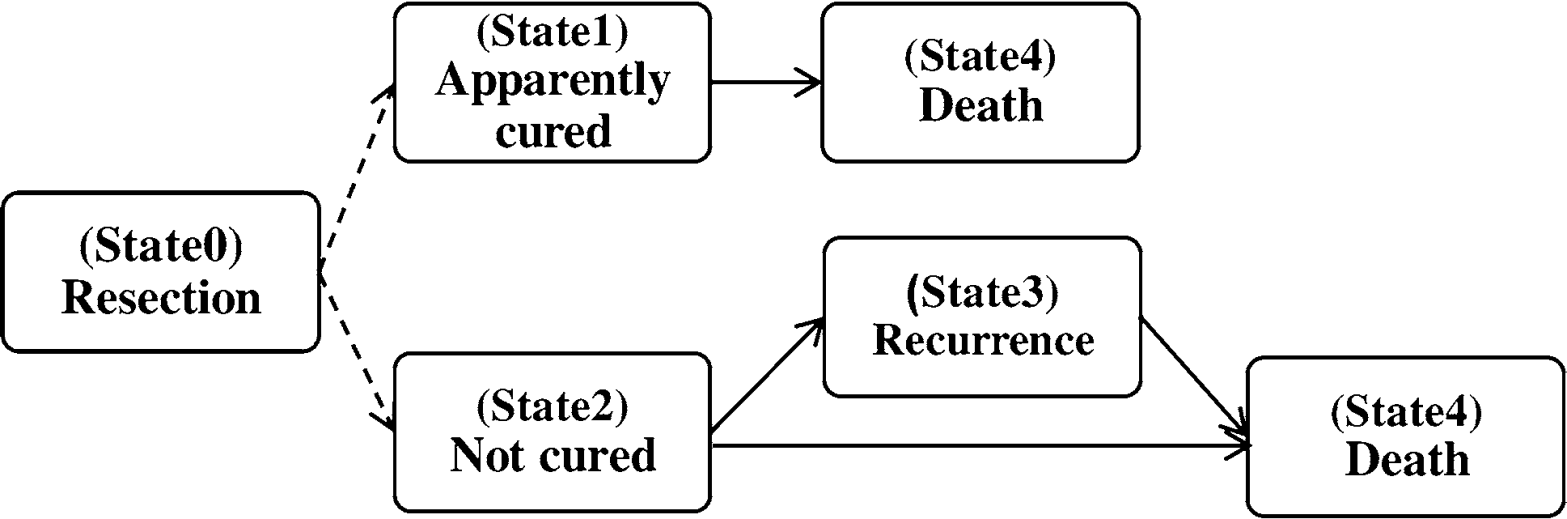
Multi-state cure model structure. The arrows show the directions of possible transitions (N = 283).

According to the available information, the patients who recurred during their follow-up were assumed as not cured patients with observed recurrence time, while the patients who did not recur during their follow-up were assumed to be censored for recurrence. Apparently, the cured patients would have never recurred even if they had been followed longer. Each patient can transient to death state either with or without a prior recurrence. So, the multi-state model (shown in Fig. 4) consisted of four states (apparently cured, not cured, recurrence, and death) and then there were four transitions between these states including: (1) the transition from not cured group to death, (2) the transition from apparently cured group to death, (3) the transition from not cured group to recurrence, and (4) the transition from recurrence to death. Patients with unknown exact time of death were considered as censored. As shown in Fig. 4, there were two latent states that each patient was assigned to one of them based on her/his information.

In the standard mixture formulation of the cure model, when there is one event of interest, the marginal survival function, $S(t)$, is given by $S(t) = P + (1 - P)S_{0} (t)$, where $S_{0} (t)$ is the conditional survival function for the uncured group and the survival for the cured group is equal to 1. P shows the proportion of the population who has never experienced the event of interest (in this case CRC recurrence). Here P provides information about the tumor and the effect of the treatment on cell killing.

A logistic model, $Logit(p(NonCure = 1|X_{i} )) = \alpha + \gamma X_{i}$, is used to describe cured fraction, where $X_{i}$ is the vector of the covariates associated with the vector of coefficients $\gamma$ and $\alpha$ is the intercept term^27^.

The distributions of event times given cured status are described by proportional hazards models. The hazard for transition from state k to state j for the ith subject based on proportional hazards model is defined as,

1 $$\lambda_{jk} (t_{i} ;X_{i} ) = \lambda_{jk}^{0} (t_{i} )\exp (\beta_{jk}^{T} X_{i} ),$$

where $X_{i}$ is the vector of covariates that their effects (their associated coefficients $\beta_{jk}$ for transition from state j to state k) can change over states and $\lambda_{jk}^{0} (t_{i} )$ is the baseline hazard for subject i for transition from state j to state k at time t.

Then the survival distribution for each transient state is constructed by $\lambda_{jk} (t_{i} )$ as follows;

2 $$\begin{aligned} & S_{1} (t) = \exp ( - \int\limits_{0}^{t} {\lambda_{14} (u)du} ) \\ & S_{2} (t) = \exp ( - \int\limits_{0}^{t} {\lambda_{23} (u)du - \int\limits_{0}^{t} {\lambda_{24} (u)du} } ) \\ & S_{3} (t|t_{r} ) = \exp ( - \int\limits_{0}^{{t - t_{r} }} {\lambda_{34} (u)du} ), \\ \end{aligned}$$

where $t_{r}$ is the entry time into state 3.

Here, it was assumed that the multi-state process is a continuous-time discrete-state semi-Markov process, in which the clock set back to 0 at the time of entry into a new state. The term continuous time refers to the fact that the process could switch from each state to the other states at any time and the term discrete-state means that the state space is finite. So, a semi-Markov multi-state model is used to model the transition between different states.

For transition 1 → 4 and 2 → 4, $t_{i}$ is the death or censored time, for transition 2 → 3, $t_{i}$ is the recurrence or censored time, and for transition 3 → 4, $t_{i}$ is the gap time between entry into state 3 and state 4 (i.e. $t - t_{r}$) proportional hazard regression was used to model the residual time in state 3 before entering state 4 as follow:

3 $$\lambda_{34} (t - t_{ir} ) = I(t > t_{ir} )\lambda_{34}^{0} (t - t_{ir} )\exp (\beta_{34}^{T} X_{i} ).$$

In addition, for the transition from state 3 to state 4, the time-to-recurrence (sojourn time in the recurrence state) is considered as a covariate in addition to other covariates. All baseline hazards are assumed unrestricted (i.e. the equality of baseline hazards for transition 1 → 4 and 2 → 4 was not compulsory).

At the baseline, all patients who experience recurrence were known to be not cured and all other patients have unknown cure status. Although it has been never known for sure that patients are cured, it may be believed that patients who are still at risk after a specified time are cured^29^. Here, Subjects still at risk for recurrence and death after 50 months were assumed to be apparently cured.

It should be noted that the parameters of both multi-state and logistic models (included in the multi-state cure model) are estimated jointly via a joint likelihood function which can be found in Conlon et al.^28^. An EM algorithm proposed by Lauren et al.^29^ was used to estimate the parameters of the model. The variables were selected based on the univariate model, i.e. at each time, a univariate model (a multi-state cure model which contains only one predictor variable) was fitted. Then, the variables which was significant at the level of 0.05 on at least one of the transitions have included in the final multiple multi-state cure model. However, for the transition from recurrence to death state, the model was faced with the problem of non-convergence due to the small sample size. To solve this issue, we included only the clinically important variables in the multi-state cure model for this transition. All statistical analyses were performed at a significance level of 0.05 using the MultiCure package of R software, version 3.5.3 (The R Foundation for Statistical Computing, Vienna, Austria, RC Team. URL http://www.R-project.org).

## Supplementary Information

Supplementary Information.

## Data Availability

The datasets analyzed during the current study are available from the corresponding author on reasonable request with permission of MA.
