# Supplementary figures and images for: Joint modelling of colorectal cancer recurrence and death after resection using multi-state model with cured fraction
Source: Sci Rep. 2021 Jan 13;11:1016. doi: 10.1038/s41598-020-79969-6 (PMC7806811; doi:10.1038/s41598-020-79969-6)

**
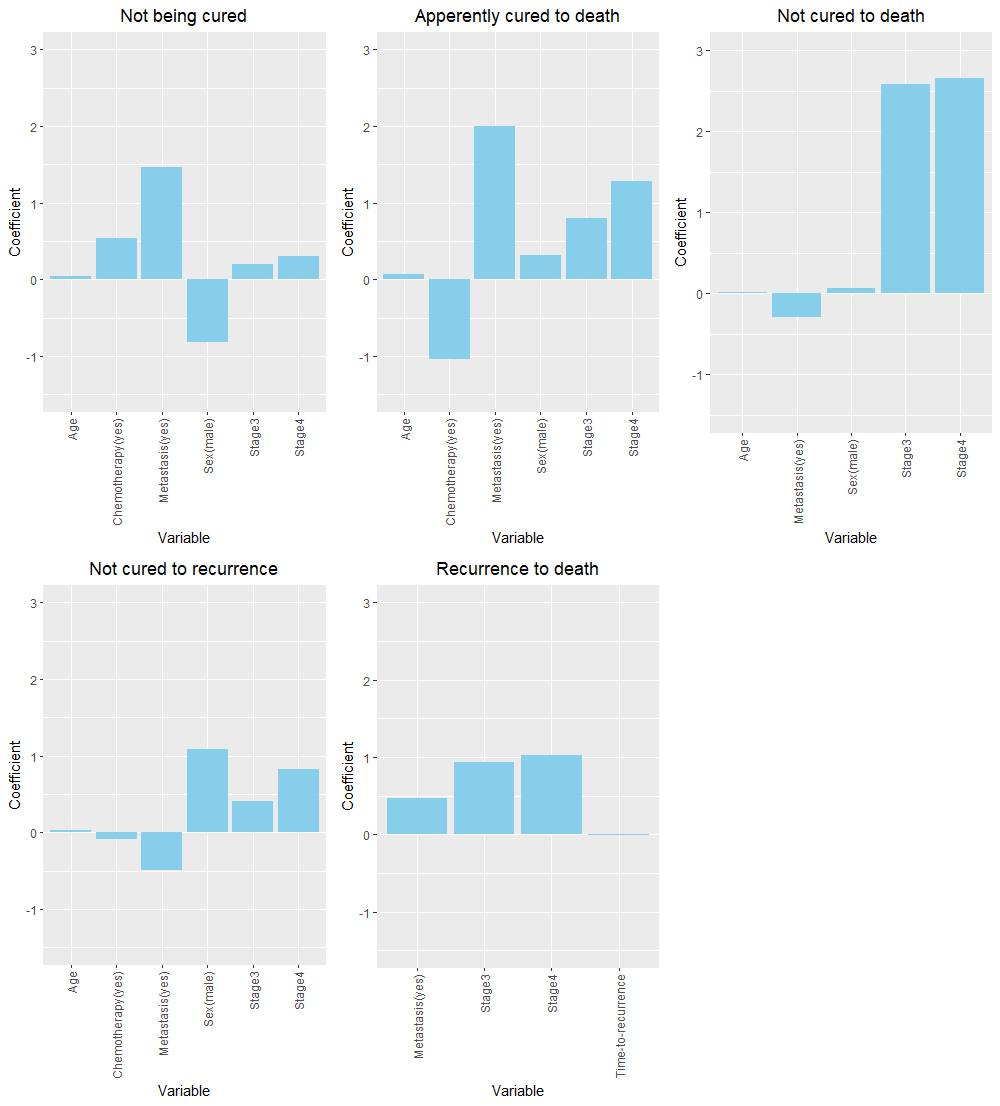
**

**Figure A.** The effects of applied variables on different parts of the multi-state cure model.

Supplement: Supplementary file 1 — Supplementary Information. [file 41598_2020_79969_MOESM1_ESM.docx]
